# Gene expression data from acetaminophen-induced toxicity in human hepatic *in vitro* systems and clinical liver samples

**DOI:** 10.1016/j.dib.2016.03.069

**Published:** 2016-03-26

**Authors:** Robim M. Rodrigues, Olivier Govaere, Tania Roskams, Tamara Vanhaecke, Vera Rogiers, Joery De Kock

**Affiliations:** a Department of *in vitro* Toxicology and Dermato-Cosmetology, Faculty of Medicine and Pharmacy, Vrije Universiteit Brussel, Laarbeeklaan 103, 1090 Brussels, Belgium; b Translational Cell & Tissue Research Unit, Department of Imaging & Pathology, Katholieke Universiteit Leuven (KUL), Minderbroedersstraat 12, 3000 Leuven, Belgium

**Keywords:** Transcriptomics, *in vitro*, Liver toxicity, Acetaminophen

## Abstract

This data set is composed of transcriptomics analyses of (i) liver samples from patients suffering from acetaminophen-induced acute liver failure (ALF) and (ii) hepatic cell systems exposed to acetaminophen and their respective controls. The *in vitro* systems include widely employed cell lines *i.e.* HepaRG and HepG2 cells as well as a novel stem cell-derived model *i.e.* human skin-precursors-derived hepatocyte-like cells (hSKP-HPC). Data from primary human hepatocytes was also added to the data set “Open TG-GATEs: a large-scale toxicogenomics database” (Igarashi et al., 2015) [1]. Changes in gene expression due to acetaminophen intoxication as well as comparative information between human *in vivo* and *in vitro* samples are provided. The microarray data have been deposited in NCBI׳s Gene Expression Omnibus and are accessible through GEO Series accession number GEO: GSE74000. The provided data is used to evaluate the predictive capacity of each hepatic *in vitro* system and can be directly compared with large-scale publically available toxicogenomics databases. Further interpretation and discussion of these data feature in the corresponding research article “Toxicogenomics-based prediction of acetaminophen-induced liver injury using human hepatic cell systems” (Rodrigues et al., 2016) [2].

**Specifications Table**

**Table:** 

Subject area	*Biology*
More specific subject area	*Toxicology*
Type of data	*Tables and Figures*
How data was acquired	*Amplified RNA fragments were hybridized to Affymetrix Human Genome U133 plus 2.0 array and scanned with Affymetrix Gene-Chip Scanner-3000-7G. Background correction, summarization (median polish) and normalization (quantile) were done by Robust Multi-array Analysis (RMA) using RMAExpress v1.0.5 software. Ingenuity Pathway Analysis software (version December 2014) was used for functional analyses and set-up of predictive diagrams.*
Data format	*RAW/Analyzed*
Experimental factors	*All hepatic in vitro models were exposed to their corresponding acetaminophen-based IC*_*10*_*concentrations and vehicle controls 24 hours prior to sampling. Acetaminophen-based cytotoxicity was first assessed in vitro using the MTT viability assay. The 24 hour IC*_*10*_*value for each cell type (HepG2, HepaRG and hSKP-HPC) was determined by 4-parameter logistic nonlinear regression analysis of the obtained dose response curves.*
Experimental features	*Total RNA was extracted from samples exposed to acetaminophen (IC*_*10*_*) and their respective vehicle controls as well as from clinical samples of acetaminophen-induced acute liver failure (ALF) and healthy livers. RAW transcriptomics data were generated by Affymetrix microarray technology. Changes in gene expression due to acetaminophen-induced toxicity for each of the hepatic in vitro models as well as for clinical samples of ALF were calculated as fold change of their respective vehicle controls. Data obtained from all hepatic in vitro models exposed to acetaminophen (IC*_*10*_*) were compared to clinical samples of acetaminophen-induced ALF. Ingenuity Pathway Analysis software was used to build an ALF prediction diagram and to evaluate the predictive capacity of each hepatic in vitro system.*
Data source location	*Department of in vitro Toxicology and Dermato-Cosmetology (IVTD), Vrije Universiteit Brussel (VUB), Brussels, Belgium*
Data accessibility	*Data is within this article. RAW microarray data have been deposited in NCBI׳s Gene Expression Omnibus and are accessible through GEO Series accession number GEO: GSE74000.*

**Value of the data**

• These data can be used as a reference data set for predictive analysis of acetaminophen-induced liver injury.
• Transcriptomics datasets were obtained from scarcely available liver samples of patients suffering from acetaminophen-induced acute liver failure as well as from healthy individuals. They are mostly of interest for the scientific community working on human hepatic toxicity.
• Transcriptomics data of the most widely employed hepatic *in vitro* systems (exposed to acetaminophen and corresponding controls) are also provided. These data can be used in comparative studies in which other hepatic cell systems are used.

## 1 Data

Gene expression data obtained by microarray are provided from (1) clinical samples obtained from patients suffering from acetaminophen-induced acute liver failure and healthy liver samples and from (2) the most widely employed hepatic systems (HepaRG cells, HepG2 cells and primary human hepatocytes) as well as a recently introduced stem cell-based hepatic model (human skin-precursors-derived hepatocyte-like cells (hSKP-HPC)) exposed to acetaminophen (and respective controls). All data were obtained using whole genome microarray arrays (Affymetrix Human Genome U133 plus 2.0).

## 2 Experimental design, materials and methods

Samples were isolated from either clinical liver samples from patients suffering from acetaminophen-induced acute liver failure (ALF) and samples of healthy livers or various hepatic *in vitro* models, *i.e.* HepaRG, HepG2 and hSKP-HPC. Corresponding vehicle controls were also included in the analysis [2].

The following *in vitro* and *in vivo* samples were analyzed:

(1) HepG2 cells exposed to IC_10_ concentration (2 mM) of acetaminophen and untreated controls: *n*=3 biological replicates for each condition.
(2) HepaRG cells exposed to IC_10_ concentration (13 mM) of acetaminophen and untreated controls: *n*=3 biological replicates for each condition.
(3) hSKP-HPC exposed to IC_10_ concentration (18 mM) of acetaminophen and untreated controls: *n*=3 biological replicates for each condition: prior to acetaminophen exposure, hSKP were isolated, cultured and differentiated into hSKP-HPC as previously described [3], [4].
(4) Primary human hepatocytes exposed to IC_10_ concentration (5 mM) of acetaminophen and untreated controls available through Open TG-GATEs database: *n*=2 biological replicates for each condition [1].
(5) Clinical samples of patients suffering from acetaminophen-induced acute liver failure and healthy liver samples: *n*=3 biological replicates for ALF samples and *n*=2 biological replicates for healthy liver.

Total RNA isolation was conducted using the GenElute Mammalian Total RNA Purification Miniprep Kit (Sigma-Aldrich) following the manufacturer׳s instructions.

Total RNA (100 ng) was used for amplification and *in vitro* transcription using the Genechip 3’ IVT Express Kit following the manufacturer׳s instructions (Affymetrix). The amplified RNA was purified with magnetic beads and 15 μg Biotin-aRNA was treated with fragmentation reagent. 12.5 μg fragmented aRNA was hybridized to Affymetrix Human Genome U133 plus 2.0 arrays along with a hybridization cocktail solution and then placed in a Genechip Hybridization Oven-645 (Affymetrix) rotating at 60 rpm at 45 °C for 16 h. After incubation, arrays were washed on a Genechip Fluidics Station-450 (Affymetrix) and stained with the Affymetrix HWS kit as indicated by the manufacturer׳s protocols (Affymetrix).

The chips were scanned with an Affymetrix Gene-Chip Scanner-3000-7G and the quality control matrices were confirmed with the Affymetrix GCOS software following the manufacturer׳s guidelines. Background correction, summarization, and normalization of all data were done with Robust Multiarray Analysis (RMA). Changes in gene expression due to acetaminophen-induced toxicity for each of the hepatic *in vitro* models as well as for clinical samples of ALF were calculated as fold change of their respective controls. Ingenuity Pathway Analysis software (version December 2014; Qiagen) was used to build an ALF prediction diagram based on the microarray data of patients suffering from acetaminophen-induced liver failure. This was then used to evaluate the predictive capacity of each hepatic *in vitro* system by comparing the acetaminophen-induced gene expression modulation to clinical samples of acetaminophen-induced ALF.

**Fig. 1:**
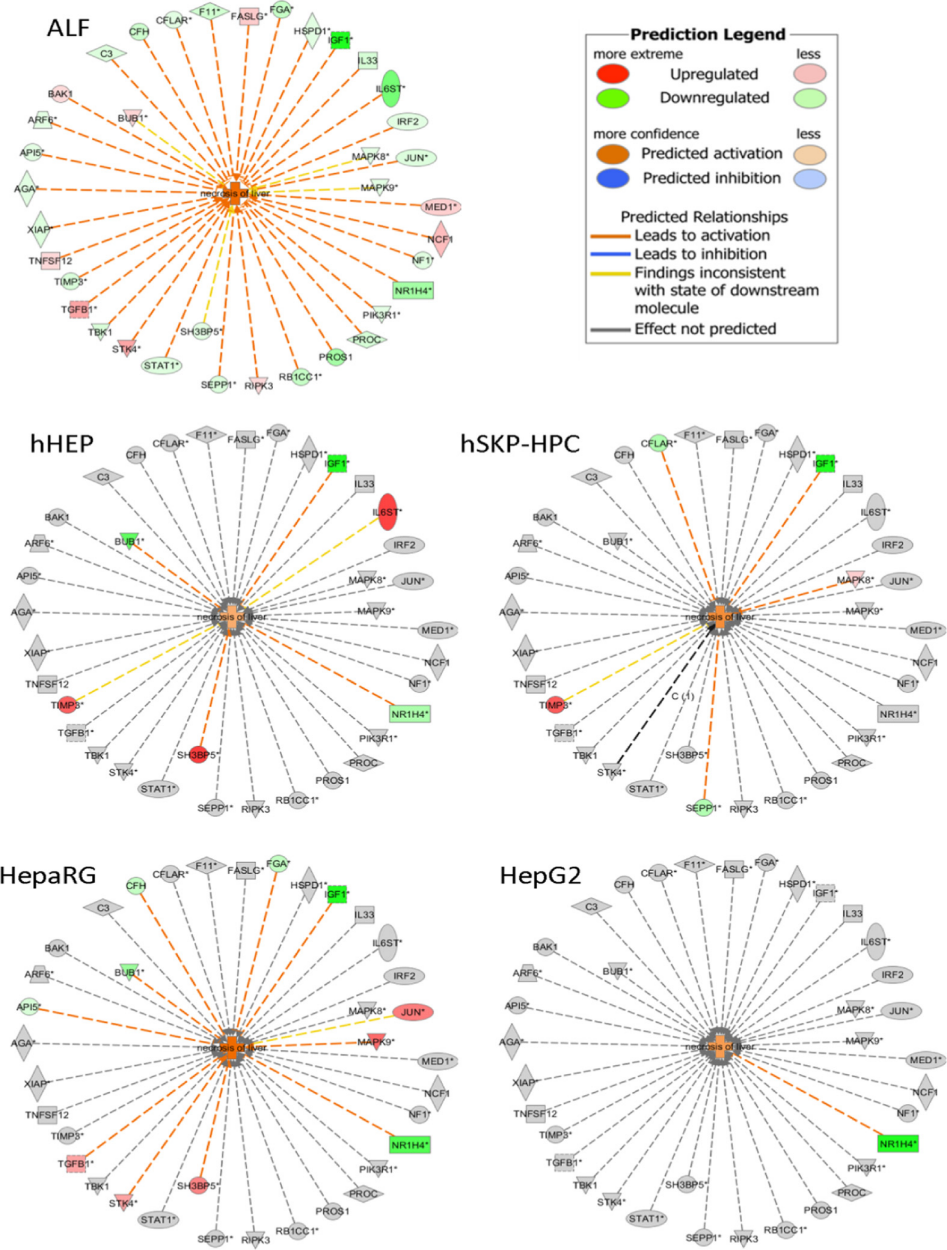
Prediction diagram for the toxicity gene class “Necrosis of Liver”. The selection of genes was based on the significantly modulated molecules of this gene class in the ALF samples *versus* control samples. For this selection of genes, the “Necrosis of Liver” function was activated for hHEP, HepaRG and hSKP-HPC, but not for HepG2.

In Fig. 1, the data are shown of the four investigated hepatic *in vitro* models in which gene modulation due to acetaminophen-induced toxicity was assessed. It depicts a prediction diagram for the toxicity gene class “Necrosis of Liver”. Table 1 shows a selection of genes corresponding to the “Damage of Liver” gene class with separate gene expression modulation values for each investigated hepatic system. This data accompanies the prediction chart “Damage of Liver” published in the research article complimentary to the current *data in brief* article [1]. Table 2 shows a selection of genes corresponding to the “Necrosis of Liver” prediction chart indicated in Fig. 1. The selection of the genes in Table 1, Table 2 as well as the corresponding prediction charts were based on the differential gene expression between the liver samples of patients suffering from acetaminophen-induced liver failure and samples obtained from healthy livers. The cut-off was set at minimum 2-fold expression divergence and statistical significance with *p*-value <0.05 (Fisher׳s exact).

## Figures and Tables

**Table 1:** Modulation of genes (fold change relative to respective controls) of the “Damage of Liver” gene class selected for APAP-induced ALF samples.

**Damage of liver genes**	**ALF**	**hSKP-HPC**	**hHEP**	**HepaRG**	**HepG2**
NCF1	3.6	−1.1	−1.0	1.1	−1.0
FASLG	2.9	−1.1	−1.3	1.1	−1.3
MED1	2.7	−1.4	1.2	1.3	1.2
CD44	2.1	2.4	3.2	3.0	3.2
SH3BP5	−2.0	1.6	3.0	2.6	3.0
FLT1	−2.6	2.2	1.6	−1.2	1.6
CTNNB1	−3.6	1.7	−1.2	−1.3	−1.2
ALB	−3.7	8.1	−1.7	−7.4	−1.7
SMAD4	−3.7	−1.8	1.5	1.5	1.5
PAWR	−3.8	−2.7	1.3	−2.6	1.3
IFNGR1	−3.9	1.3	1.5	−2.4	1.5
BPNT1	−4.2	−1.1	−2.1	−1.8	−2.1
TLR3	−4.5	−1.3	−3.6	−21.6	−3.6
CFH	−5.6	−1.0	−1.4	−3.1	−1.4
EGR1	−7.2	33.9	2.3	6.1	2.3
RDX	−8.7	1.3	1.6	1.1	1.6
SERPING1	−10.1	−1.1	−1.0	−1.3	−1.0
GCLC	−11.8	2.8	2.7	1.4	2.7
IL6ST	−14.3	1.5	2.9	1.3	2.9
SAA2	−16.1	−4.0	1.0	−1.4	1.0
IGF1	−104.2	−48.5	−7.0	−16.0	−7.0

**Table 2:** Modulation of genes (fold change relative to respective controls) of the “Necrosis of Liver” gene class selected for APAP-induced ALF samples.

**Liver necrosisgenes**	**ALF**	**hSKP-HPC**	**hHEP**	**HepaRG**	**HepG2**
**TGFB1**	4.82	1.20	3.50	2.01	1.41
**STK4**	4.80	1.83	1.43	2.02	1.39
**NCF1**	3.62	−1.07	−1.00	1.14	1.01
**FASLG**	2.91	−1.07	−1.26	1.08	−1.14
**MED1**	2.69	−1.38	1.20	1.26	−1.06
**BUB1**	2.47	1.89	−4.12	−4.89	1.20
**TNFSF12**	2.25	−1.73	−1.36	−1.04	−1.11
**RIPK3**	2.24	−1.30	−1.35	−1.13	−1.04
**BAK1**	2.12	−1.05	1.05	1.26	1.21
**SH3BP5**	−2.04	1.56	2.95	2.62	1.68
**API5**	−2.16	1.20	1.92	−2.10	−1.16
**MAPK9**	−2.53	−1.21	1.91	3.17	1.24
**IRF2**	−2.67	1.29	−1.28	1.01	−1.04
**ARF6**	−2.75	−1.44	1.19	−1.44	1.15
**HSPD1**	−2.88	1.92	−1.53	1.27	−1.60
**AGA**	−2.89	−1.21	1.19	−1.11	1.58
**MAPK8**	−3.29	2.82	1.73	1.88	1.41
**CFLAR**	−3.49	−2.66	−1.72	−1.82	−1.57
**PIK3R1**	−3.52	−1.27	1.31	1.35	1.19
**NF1**	−3.66	−1.77	1.22	1.28	1.28
**STAT1**	−3.81	1.50	−1.34	−1.35	1.51
**XIAP**	−3.92	−1.38	1.50	−1.50	−1.14
**JUN**	−3.94	1.91	−1.44	2.77	1.25
**C3**	−4.04	−1.07	−1.56	−1.30	−1.13
**SEPP1**	−4.04	−2.57	−1.72	−1.73	−1.24
**TIMP3**	−4.43	8.65	2.47	1.48	−1.30
**TBK1**	−4.50	−1.86	1.04	1.24	1.03
**IL33**	−4.66	−1.73	1.14	−1.39	1.02
**PROC**	−4.84	1.01	−1.80	−1.16	−1.26
**F11**	−5.20	−1.13	−1.53	−1.68	−1.14
**CFH**	−5.61	−1.02	−1.40	−3.10	1.13
**FGA**	−6.35	−1.10	−1.65	−3.13	1.62
**RB1CC1**	−6.44	1.55	1.50	1.26	1.18
**PROS1**	−9.55	−1.14	−1.66	−1.71	−1.11
**NR1H4**	−9.81	1.21	−2.26	−8.36	−2.57
**IL6ST**	−14.33	1.50	2.88	1.25	−1.24
**IGF1**	−104.24	−48.52	−6.98	−15.97	−1.17
