# Supplementary material for: Gene expression data from acetaminophen-induced toxicity in human hepatic in vitro systems and clinical liver samples
Source: Data Brief. 2016 Mar 26;7:1052–7. doi: 10.1016/j.dib.2016.03.069 (PMC5063792; doi:10.1016/j.dib.2016.03.069)
Supplement: Supplementary file 1 — Supplementary material [file mmc1.doc]

There are no conflicting interests.
